# Self-Assembled Cationic-Covered Nanoemulsion as A Novel Biocompatible Immunoadjuvant for Antiserum Production Against *Tityus serrulatus* Scorpion Venom

**DOI:** 10.3390/pharmaceutics12100927

**Published:** 2020-09-29

**Authors:** Arthur Sérgio Avelino de Medeiros, Manoela Torres-Rêgo, Ariane Ferreira Lacerda, Hugo Alexandre Oliveira Rocha, Eryvaldo Sócrates Tabosa do Egito, Alianda Maira Cornélio, Denise V. Tambourgi, Matheus de Freitas Fernandes-Pedrosa, Arnóbio Antônio da Silva-Júnior

**Affiliations:** 1 Laboratory of Pharmaceutical Technology and Biotechnology, Department of Pharmacy, Federal University of Rio Grande do Norte-UFRN, Natal 59010-180, Brazil; arthursergiomedeiros@gmail.com (A.S.A.d.M.); manoelatorres@ufrn.edu.br (M.T.-R.); arianelacerda@yahoo.com.br (A.F.L.); socratesegito@gmail.com (E.S.T.d.E.); 2 Graduate Program of Chemistry, Chemistry Institute, Federal University of Rio Grande do Norte, Avenue Senador Salgado Filho, 3000, Lagoa Nova, Natal 59072-970, Brazil; 3 Department of Biochemistry, Federal University of Rio Grande do Norte-UFRN, Natal 59010-180, Brazil; hugo@cb.ufrn.br; 4 Department of Morphology, Federal University of Rio Grande do Norte-UFRN, Natal 59010-180, Brazil; aliandamaira@gmail.com; 5 Laboratory of Immunochemistry, Butantan Institute, Av. Vital Brasil, 1500, São Paulo 05503-900, Brazil; denise.tambourgi@butantan.gov.br

**Keywords:** *Tityus serrulatus* antiserum, nanoemulsion adjuvant, liquid crystal, phase transition, surface functionalization, immunoadjuvant

## Abstract

This study assesses the efficacy of different nanoemulsion formulations as new and innovative adjuvants for improving the in vivo immunization against the *Tityus serrulatus* scorpion venom. Nanoemulsions were designed testing key-variables such as surfactants, co-solvents, and the influence of the temperature, which would be able to induce the phase transition from a liquid crystal to a stable nanoemulsion, assessed for four months. Additionally, cationic-covered nanoemulsion with hyper-branched poly(ethyleneimine) was prepared and its performance was compared to the non-cationic ones. The physicochemical properties of the selected nanoemulsions and the interactions among their involved formulation compounds were carefully monitored. The cytotoxicity studies in murine macrophages (RAW 264.7) and red blood cells were used to compare different formulations. Moreover, the performance of the nanoemulsion systems as biocompatible adjuvants was evaluated using mice immunization protocol. The FTIR shifts and the zeta potential changes (from −18.3 ± 1.0 to + 8.4 ± 1.4) corroborated with the expected supramolecular anchoring of venom proteins on the surface of the nanoemulsion droplets. Cell culture assays demonstrated the non-toxicity of the formulations at concentrations less than 1.0 mg/mL, which were able to inhibit the hemolytic effect of the scorpion venom. The cationic-covered nanoemulsion has shown superior adjuvant activity, revealing the highest IgG titer in the immunized animals compared to both the non-cationic counterpart and the traditional aluminum adjuvant. In this approach, we demonstrate the incredible potential application of nanoemulsions as adjuvants, using a nanotechnology platform for antigen delivery system on immune cells. Additionally, the functionalization with hyper-branched poly(ethyleneimine) enhances this recognition and improves its action in immunization.

## 1. Introduction

Scorpion sting is the most common and primary cause of death caused by accidents with venomous animals in tropical and sub-tropical countries [1]. In Brazil, the most severe accidents caused by scorpion occurs with the species *Tityus serrulatus* [2]. On the other hand, the antiserum therapy is the only approved and available treatment for accidents with venomous animals such as spiders, snakes, and scorpions. These immunotherapeutic products are produced by the immunization of horses, by injecting the specific venom associated with a suitable traditional adjuvant. However, most of the antibodies presented in these antiserum products are non-specific against the toxins from venom animals, and they may cause side effects and present low potency.

*Tityus serrulatus* venom (TsV) presents a complex and rich composition, which includes oligopeptides, glycosaminoglycans, proteins, nucleotides, amino acids, and a vast variety of peptides. Some of these compounds are capable to modulate ionic channels such as Na^+^, K^+^, Ca^2+^, and Cl^−^, with enzymes activity, mainly neurotoxins [3,4]. The crude venom can be easily dispersed in water or saline solutions, preserving the stability of their ionic peptides, which can be purified and the enzymes can be separated by size exclusion or ion exchange chromatography [5]. Thus, the use of adjuvants able to control the release of TsV can offer several benefits in the immunization. The peptides and proteins can be anchored on the surface of particles by electrostatic and hydrophobic interactions [6,7,8].

Traditional adjuvants have been used for centuries in vaccines and for anti-serum production for improving the adaptive immune response against antigens of harmful pathogens and toxins. However, although the list of diseases that are included in the immunization programs of the World Health Organization (WHO) is quite extensive [9], the number of approved adjuvants for human use is very limited. The adjuvants most used include aluminum salts, emulsions, liposomes, and agonists for toll-like receptor (TLR) [10,11]. Among them, the aluminum-based salt was the pioneer compound used as an adjuvant for human vaccines [11,12]. However, it presents major drawbacks such as lack of specificity of its elicited immune response, safety problems, and side effects. These limitations are generally related to the physicochemical or biological properties of the aluminum-based salt adjuvant. Additionally, a suitable antigen-release rate is desired for improving the antigen-specific cellular and humoral response [13].

Nanotechnology platforms, such as nanoparticles, nanoemulsions, liposomes, and virus-like particles [11,13], can be perfectly used to solve the drawbacks related to the adjuvants. Nanoemulsions and nanoparticles have proven to enhance antigen recognition by immune cells [14,15]. These systems potentially increase the adjuvant efficiency regarding to the expected immune response. Specifically, the nanoemulsions can induce a Th1 response due to the enhanced CpG agonist of toll like receptor (TLR) [14] and glucopyranosyl lipid [16], both agonists adjuvants that induces Th1 responses for influenza and Ebola vaccines, respectively. Particle-based adjuvants can induce the innate immunity activation through patterns receptor recognition (PRRs), which are receptors that recognize the secondary danger-associated signals. This pattern of response was previously demonstrated for aluminum particles and MF59 nanoemulsions [17]. Not only the reduced and uniform sized, but also the oil type in nanoemulsions affects adjuvant efficiency [18]. The shape of the nanocarriers also affects the recognition by the antigen-presenting cells (APC) and the clearance by the lymph nodes [19].

The hydrophobicity and charge of the particle-based adjuvants also modulate the antigen adsorption, controlling its absorption, residence time and the innate immune activation [13,17,19]. Cationic charged nanoemulsions have demonstrated improved cell recognition and mucous adhesion compared to neutral and anionic counterparts. Moreover, the performance of the cationic nanoemulsion can also be improved by changing its composition, specifically its amphiphilic compounds [20]. Among the licensed nanotechnology-based vaccines for human use, the MF59 (Novartis) squalene nanoemulsion demonstrates a broad immune response [21]. Some previous studies have also reported the use of other nanoemulsion-based adjuvants [17,20,21,22,23]. However, the performance of these nanocarriers has not yet been demonstrated for antiserum production.

Considering the association of surfactant with oil in an aqueous environment, different oil in water (O/W) nanocarriers can be prepared, such as for example microemulsions, nanoemulsions, and liquid crystals. Microemulsions and nanoemulsions are isotropic submicron emulsions generally formed by oil droplets stabilized in an aqueous phase by surfactants. The first is a transparent and thermodynamically stable colloidal dispersion due to the large concentration of surfactants, while the second is stabilized by lower concentration of surfactants, forming slight turbid colloidal dispersions and thermodynamically unstable [24,25,26]. Liquid crystals (LC) are viscoelastic mesophases formed for arranged surfactants in liquid medium, which can load considerable amount of oil due to their alternated hydrophilic and lipophilic domains [26]. The LC are anisotropic systems, with exception of the cubic phases, which are isotropic and has high viscosity. The self-assembly of surfactants can be induced by changes in the temperature or solubility in the medium, forming thermotropic or lyotropic LCs, respectively [27,28].

Nanoemulsions can be prepared using high energy methods, like ultrasonication or high-pressure homogenization [29]. Strong disruptive forces are provided from mechanical devices, which break up large droplets into nano-sized oil droplets stabilized in water by suitable amount of surfactants [30,31]. Besides the several advantages presented by these methods, their scale-up process involves many difficulties to reproduce the high input of energy in the emulsification process. Thus, low energy methods can be a useful alternative to solve this problem and produce ultrafine nanoemulsions. This strategy involves self-assembly of compounds by using phase inversion methods, spontaneous emulsification, and phase transition methods. [32].

The phase transition was reported as an interesting approach for preparing nanoemulsions by simple dilution of microemulsions with water altering the surfactant arrangement in the oil/water interface [33], from transitional bicontinuous structures [34]. In anterior studies, we have reported a similar strategy to produce biocompatible O/W nanoemulsions from suitable dilutions of lyotropic lamellar LCs [35]. According the type of the used surfactant, the phase transition strategy can incorporate concepts of phase inversion temperature (PIT), phase inversion composition (PIC), and catastrophic phase inversion (CPI). The PIT method can generate small and uniform droplets by changes of spontaneous curvature of some non-ionic surfactants, induced by temperate-dependent dehydration. Liquid crystalline or bicontinous structures are formed when a near zero surfactant curvature is observed, which can changed by rapid cooling or heating steps forming kinetically stable nanoemulsions [29,36]. A similar transition can be induced by the PIC method, in which the solubility of surfactants and consequently their curvature can be changed by gradual addition of continuous phase, with specific composition [37]. The CPI method is similar to PIC method, but the second does not induces changes in the curvature of surfactant, but changing the ratio of dispersed phase until a critical point, in which the dispersed phase became the continuous phase (phase inversion) [38,39]. Also, nanoemulsions can be formed by the spontaneous emulsification, which is favored by rapidly diffusion of surfactant and/or co-solvent molecules from dispersed phase to continuous phase, leading to formation of nano-sized droplets as the diffusion occurs [40,41].

In the present study, different preparation methods were explored to produce stable nanoemulsions to be used as adjuvants for anti-serum production for the treatment of the *Tityus serrulatus* scorpion sting. Distinct compositions and formulation approaches, including the cationic nanoemulsions covered with the hyperbranched polyethyleneimine were tested, an FDA approved biocompatible material capable to enhance nucleic acids, genes and drugs delivery [42,43,44]. The physicochemical properties of all formulations were carefully followed aiming to establish a nanotechnology platform for this purpose. Moreover, the in vitro studies in cell cultures and the in vivo experiments with BALB-C mice were performed for different formulations. Different nanocarriers are described in the literature and some of them are commercially available as adjuvants. However, the traditional approved adjuvant aluminum hydroxide Al(OH)_3_ is the most used for antiserum production against venomous animals, which was selected as the control in this study.

## 2. Materials and Methods

### 2.1. Materials

The venom from *Tityus serrulatus* scorpion (TsV) was donated by the Instituto Butantan, SP, Brazil. TsV was extracted from adult specimens of scorpions, then, lyophilized and stored at −20 °C before use. TsV solutions were prepared with PBS at the time of use. The amount of TsV was expressed by protein content, quantified by the bicinchoninic acid assay (BCA) using bovin serum albumin (BSA) as standard. The scientific use of the TsV was approved by the Brazilian Genetic Heritage Management Council (CGEN) (Process number 010843/2013-2) and registered in the National System for the Management of Genetic Heritage and Associated Traditional Knowledge (SisGen, Process N°AAF17D9). The medium-chain triglyceride (MCT) (Miglyol^®^812, Sasol^®^, Hamburg, Germany) was used as oil phase. The soy phosphatidylcholine (SPC) (purity > 95%) was purchased from Avanti Polar Lipids^®^ (Alabaster, AL, USA). The polysorbate 80 (T80), Tween^®^ 80, glycerol, propylene glycol, and octylphenol ethoxylate (Triton^®^) were purchased from Sigma-Aldrich Co. (St. Louis, MO, USA). *N*-methyl-pyrrolidone (99%) was purchased from Vetec (Duque de Caxias, RJ, Brazil) and used as co-solvent (CO). Poloxamer 188 and poloxamer 407 were purchased from Sigma-Aldrich Co. (St. Louis, MO, USA). Poly(ethyleneimine) hyper-branched 25,000 Da (PEI) was purchased from Sigma-Aldrich Co. (St. Louis, MO, USA). PBS buffer (pH = 7.4) was prepared with the following constituents: 137 mM NaCl, 3 mM KCl, 15 mM KH_2_PO_4_, 10 mM Na_2_HPO_4_. The purified water (0.1 μS/cm) was prepared using reverse osmosis purification equipment, Model OS50 LX, Gehaka (São Paulo, Brazil).

### 2.2. Design of Nanoemulsions and Liquid Crystals

#### 2.2.1. Preparation of Emulsions

Initial trials were performed using different surfactants to stabilize the MCT, as the oil phase, in water. Emulsions containing 10% of MCT oil phase, 5% of different surfactant blends and 85% of purified water (10:5:85, *w*/*w*/*w*) were prepared by using the phase inversion emulsification. The aqueous phase was added to the oil phase composed of the surfactants dispersed in the MCT oil in a thermostatic bath stabilized at 70 ± 2 °C, under magnetic stirring at 360 rpm. The composition of the surfactant blend was adjusted using three distinct hydrophilic surfactants, polysorbate 80 (T80), poloxamer 188 (PO188), poloxamer 407 (PO407), and the hydrophobic surfactant, soybean phosphatidylcholine (SPC). The surfactants were also tested in pairs, such as SPC:T80, SPC:PO188, and SPC:407 at several *w*/*w* ratios. All formulations were observed for 7 days and any instability, such as creaming, and phase separation, was recorded. The creaming index was calculated according to the following equation:

CI % = 100 × H_u_/H_T_ (1)

where CI % is the percentage of the creaming index, Hu is the height of the upper phase of the emulsion and H_T_ is the total height of the emulsion placed in the test tube.

The turbidity of the diluted samples with purified water (1:20 *v/v*) was measured at 860 nm, using a 1 cm^3^ glass cell in a UV/Visible spectrophotometer (Thermo Scientific, EvolutionTM 60S, Waltham, MA, USA). The suitable composition able to induce the formation of stable emulsion with lowest creaming index and highest turbidity of the aqueous phase was selected for assessing the effect of the co-solvent.

The addition of 10% *w*/*w* of co-solvent at the oil phase was evaluated to prevent phase separation, as we previously studied this effect [26] and considering the co-solvent influence at nanoemulsions formation and stability [45]. Three different co-solvents were tested, such as the *N*-methyl-pyrrolidone (NMP), glycerol, and propylene glycol.

#### 2.2.2. Pseudo-Ternary Phase Diagrams (PTPDs)

The mixture composed of SPC, T80, and NMP (3:1:3 *w*/*w*/*w*) was used as surfactant mixture (SM) selected from initial trials with surfactant blends and co-solvent amount to disperse oil/surfactant mixture. The surfactant mixture was mixed with the oil phase (SM/oil) at a ratio ranging from 0.1 to 0.9 *w*/*w*. To build the first PTPD the SM/oil phase was titrated with purified water for the emulsion production, under magnetic stirring at 360 rpm for 10 min, at 70 ± 2 °C.

A second PTPD was constructed, in which the mixture composed of SPC, T80, and NMP (6:2:15 *w*/*w*/*w*) was used as the surfactant mixture (SM), and, then, mixed with the SM/oil phase at the ratio ranging from 0.1 to 0.9 *w*/*w*. The aqueous phase was dripped (3 mL/min) into the oil phase at 25 ± 2 °C under magnetic stirring at 360 rpm for 10 min. An additional stirring was performed using the ultra-turraxT25 (IKA^®^, Staufen, Germany) at 11,000 rpm for 10 min.

#### 2.2.3. Droplet Size and Zeta Potential Measurements and Identification of Liquid Crystals

The mean droplet and size distribution (polydispersity index, PdI) were measured by dynamic light scattering (DLS) using Zetasizer Nano (Zetasizer Nano ZS, Malvern Instruments Ltd., Malvern, England) at 659 nm with a scattering angle of 90°, at 25 °C. The tested nanoemulsion formulations were diluted at 1:100 (*v/v*) with purified water to ensure dispersions within suitable experimental range (100–500 kcps). The zeta potential of the particles was measured by laser Doppler anemometry using the same apparatus. The data were expressed as mean ± standard deviation (SD).

#### 2.2.4. Polarized Light Microscopy (PLM) and Identification of Liquid Crystals

Samples were examined under cross-polarized light microscopy. The Leica DM 750 (Leica Microsystems GmbH, Wetzlar, Germany) equipped with a digital camera was used to analyze several fields of each sample at room temperature. The gel-like samples were selected for the polarized optical microscopy to observe any anisotropic behavior. All samples were placed on a glass slide and covered with a coverslip. Then the slide was placed on the microscope stage and polarized light was passed through the sample and then, the images were captured. The isotropic or anisotropic behavior of the samples was observed in the images taken at 100× and 400× magnification.

#### 2.2.5. Preparation of Cationic Hyper-Branched Poly(Ethyleneimine) (PEI) Covered Nanoemulsions

The gel-like samples identified as liquid crystal (anisotropic system) were subjected to a controlled dilution to obtain stable and small droplet-sized nanoemulsions (isotropic systems). For the cationic-PEI nanoemulsions (NE-PEI), the liquid crystal (2.0 g) was dripped with 4.54 g of PEI aqueous solution (0.029% *w*/*w*) at a flow rate of 3 mL/min to a final PEI content of 0.02% *w*/*w*. The studied PEI content was varied related to the major interface component, soybean phosphatidylcholine (SPC), with different PEI:SPC ratio ranging from 1:1000 to 1:170 (*w*/*w*). The gel-like samples were diluted under magnetic stirring of 375 rpm for 20 min, at 25 ± 2 °C. The distinct composition of nanoemulsions and gel-like samples are presented in Table 1.

### 2.3. Physical Stability of NE Formulations Against pH and Saline Content

The prepared gel-like samples, the nanoemulsion (NE) and the PEI nanoemulsion (NE-PEI) were stored in hermetically sealed test tubes at 25 °C and the colloidal aspect, the droplet size, and zeta potential were recorded at specific time intervals. The selected gel-like sample identified as liquid crystal was prepared using PBS buffer pH = 7.4 as the aqueous phase. This aqueous phase was dripped (3 mL/min) into the oil phase at 25 ± 2 °C under magnetic stirring at 360 rpm for 10 min. An additional stirring was performed in the ultra-turrax at 11,000 rpm for 10 min. To obtain the NE, the liquid crystal (2.0 g) was dripped with 4.54 g of PBS buffer solution (pH = 7.4) and for NE-PEI the liquid crystal was dripped with PBS buffer solution (pH = 7.4) containing PEI at 0.016 mg/mL under magnetic stirring of 375 rpm for 20 min, at 25 ± 2 °C.

In addition, the NE was also prepared with buffer aqueous phase with pH adjustments in acidic condition (pH = 5.5) and alkaline condition (pH = 8.5) in order to evaluate the robustness of the formulation in such conditions using HCl and NaOH 0.1 M for pH adjustments. The stable formulations produced with PBS pH = 7.4 was used for further experiments to evaluate the physical-chemical properties and to perform the in vitro and in vivo experiments.

### 2.4. Preparation of TsV-Loaded Nanoemulsions

A stock solution of the crude TsV was prepared at 1mg/mL in PBS solution (pH = 7.4), dispersing 5 mg of TsV in 5 mL of PBS, and the protein content was analytically quantified by the BCA protein assay kit as the manufacturer’s recommendations (Thermo Fisher Scientific, Waltham, MA, USA). In this experiment, the total protein content was determined by copper reduction in alkaline medium, forming a complex with BCA that absorbs at 560 nm. Aliquots of these TsV stock solutions were added to the NE (NE-TsV) or to the NE-PEI (NE-PEI-TsV) to give the final TsV concentration of 0.018 mg/mL. In order to attain adsorption equilibrium, the samples were remained on magnetic stirring at 375 rpm for 1 h, in ice bath. The samples remained stored at 4 °C for six weeks and the colloidal aspect, the droplet size, and the zeta potential were recorded at specific time intervals.

### 2.5. Fourier Transformed Infrared Spectroscopy (FTIR)

The FTIR spectra of different samples were recorded in an IR Prestige-21 spectrum (Shimadzu^®^, Tokyo, Japan). One droplet of the liquid NE was spread and analyzed by ATR-FTIR. The wavenumber range of 4000–1000 cm^−1^ was analyzed, taking 16 scanning runs with 1 cm^−1^ of resolution.

### 2.6. Cell Viability and Hemolysis Tests

#### 2.6.1. Cell Culture

The murine macrophage RAW 246.7 (ATCC TIB-71) cells were gently supplied by the Laboratory of Biotechnology of Natural Biopolymers, Department of Biochemistry, Federal University of Rio Grande do Norte, Brazil. The cell cultures were maintained in Dulbecco’s modified eagle’s medium (DMEM), supplemented with 10% fetal bovine serum (FBS), 1% penicillin-streptomycin, under sterile conditions, at 37 °C, with 5% CO_2_ and a humidified atmosphere.

#### 2.6.2. MTT Reduction Assay

The cell viability assay was evaluated in the RAW 264.7 cell line using the MTT (3-methyl-[4-5- dimethylthiazol-2-yl]-2,5-diphenyltetrazolium bromide) assay reduction assay [46]. The cell line was seeded in 96-well microplates at the density of 7 × 10^3^ cells/well with 100 μL of DMEM medium, at 37 °C and 5% CO_2_, overnight. Confluent cell-monolayers contained in 96-well plates were incubated with serial dilutions of blank (PBS) and TsV-loaded NE samples (15.6 to 0.24 mg/mL of NE). The plates were incubated for 24 h at 37 °C. Thereafter, the growth medium was aspirated, and the plate incubated with 100 µL of MTT solution (259 mg/mL). The purple formazan crystals were dissolved in 100 µL of ethanol and the absorbance measured at 570 nm, using the Epoch 2 microplate spectrophotometer (Biotek^®^, Winooski, VT, USA) [46]. The PBS solution was used as a negative control for 100% of cell viability. Control and test samples were assayed in triplicate and the assay was repeated three times. The results were compared using two-way ANOVA statistical analysis and Bonferroni post-test, comparing different groups of adjuvants and, also, their different concentrations. Samples were considered non-statistical different when *p* > 0.05. The samples considered statistically different was marked with * for *p* < 0.05, ** for *p* < 0.01 and *** for *p* < 0.001.

#### 2.6.3. Hemolysis Test

Freshly collected A+ blood samples from healthy human donors from a blood bank were used, in accordance with the declaration of Helsinki and approved protocol 2.809.485 in the Ethics Committee of Research with Humans of the Onofre Lopes Hospital from the Federal University of Rio Grande do Norte (HUOL/UFRN). Each 1 mL of blood collected in K_3_EDTA (1.5 mg EDTA: 1 mL blood) was immediately used for the experiments. The blood was centrifuged at 400× *g* for 10 min in a centrifuge 5804R model (Eppendorf ^®^, São Paulo, Brazil). The plasma and white blood cell layers were removed after centrifugation. The red blood cells were then suspended to 20% suspension (*v/v*) with PBS solution, and centrifuged at 700× *g* to remove the aqueous phase, repeating the process two more times until clear red blood cell suspension was obtained. Each 1 mL of adjuvant samples were placed in the Eppendorf tubes plus 50 µL of red blood cells suspension 20%(*v/v)*) for a final concentration of 1% of red blood cells. The samples were incubated in triplicate for 1 h at 37 °C. All the adjuvant samples were solubilized in PBS to isotonic control. After incubation, the samples were, then, centrifuged at 200× *g* in the Eppendorf centrifuge 5418R model for 10 min and the supernatant was carefully removed to a 96-well plate and analyzed in the Epoch 2 microplate spectrophotometer (Biotek^®^, Winooski, VT, USA) at 540 nm wavelength. The considered positive control was an octylphenol ethoxylate solution of 1% (*v/v*) and the negative control was PBS. The hemolysis percentage was calculated according to the following Equation (2):

Hemolysis (%) = (ABS_samples_ − ABS_negative control_) *100/(ABS_positive control_ − ABS_negative control_) (2)

The results were compared using one-way ANOVA statistical analysis and Tukey’s multiple comparison test, comparing different groups of adjuvants. The samples considered statistically different was marked with * for *p* < 0.05, ** for *p* < 0.01 and *** for *p* < 0.001.

### 2.7. In Vivo Immunization Performance

#### 2.7.1. Animals

Male and female BALB/c mice, with 6–8 weeks old and weight of 25–35 g were selected for the experiments. The experimental protocol (approval code 027.040/2017) was in accordance with the Brazilian College of Animal Experimentation (Brazil, 2008), and approved by the Ethics Committee of Animals Use in Research (CEUA) of the Federal University of Rio Grande do Norte (UFRN). Each animal group were divided in 30 × 19 × 13 propylene cages (1394 cm^2^) during the experiments with maximum 5 animals per cage and 7 animals per group (3–4 animals per cage). The animals were maintained at controlled conditions of temperature (22 ± 2 °C) and luminosity (light and dark control). The animals were feed with free water and food intake (*ad libitum*), except at the day of sedation, in which they were fasted overnight and transferred for experimentation room one hour before tests for conditioning.

#### 2.7.2. Immunization Protocol

The immunization was performed by subcutaneous injection of 100 µL of scorpion venom at the mice dorsal region with selected adjuvants, previously stored at 4 °C, six times, once a week. The immunizing groups included NE, NE-PEI and Al(OH)_3_ (0.1% *w/v*) adjuvants and their associations with TsV at 9 µg/mL and 18 µg/mL, with seven (*n* = 7) animals per group. At the end of six weeks, the animals were sedated with ketamine 10% *w/v* (30 mg/kg) and xilazine 2% *w/v* (300 mg/kg) and then, euthanized with cardiac punction. After blood collection, the samples were stored for 30 min at 37 °C and for 2 h at 4 °C for clot retraction. Immediately afterward, the samples were centrifuged three times at 15,000× *g* for 5 min at 4 °C to remove the red blood cells using the centrifuge 5804R model (Eppendorf^®^, São Paulo, Brazil). The serum was stored at −20 °C until the titration of antivenom IgG by the Enzyme-Linked Immunosorbent Assay test (ELISA).

#### 2.7.3. IgG Titration by ELISA

The antivenom IgG titer of immunized animals was obtained by the ELISA titration. First, a 96 well plate for ELISA was coated with the crude TsV with 1 µg/100 µL per well, incubating overnight for 12 h at 4 °C in a wet chamber. The plate was, then, washed three times with PBS and blocked with 200 µL per well of bovine serum albumin (BSA 5% *w/v*)/PBS with 2 h incubation at 4 °C in a wet chamber. The plate was washed three times with PBS/polysorbate 20 0.05% and, then, the primary antibody from crude mice serum was added with serial dilutions from 1:100 to 1:25600 with BSA (0.1% *w/v*)/PBS, incubating for one hour at 4 °C in a wet chamber. The plate was washed three times with PBS/polysorbate 20 0.05% and then, 100 µL of marked anti-mice IgG was added and incubated for one hour at 37 °C in a wet chamber. The plate was washed three times with PBS/polysorbate 20 0.05% and then, revealed with 50 µL/well of peroxidase substrate. The plate was, then, incubated in the dark for 15 min and the stop solution containing 50 µL/well of H_2_SO_4_ 4N was added. The absorbance results were taken in the Epoch 2 microplate spectrophotometer (Biotek^®^, Winooski, VT, USA) at 490 nm. The titers were calculated as the maximum dilution in which the optical density of samples from immunized animals with TsV was, at least, twice the optical density of animals immunized with adjuvant only.

### 2.8. Statistical

The titer results were compared using one-way ANOVA statistical analysis and Tukey’s multiple comparison test, comparing different groups of adjuvants. The samples considered statistically different was marked with * for *p* < 0.05, ** for *p* < 0.01 and *** for *p* < 0.001.

## 3. Results

### 3.1. Design of Nanoemulsions and Liquid Crystal

**Figure 1:**
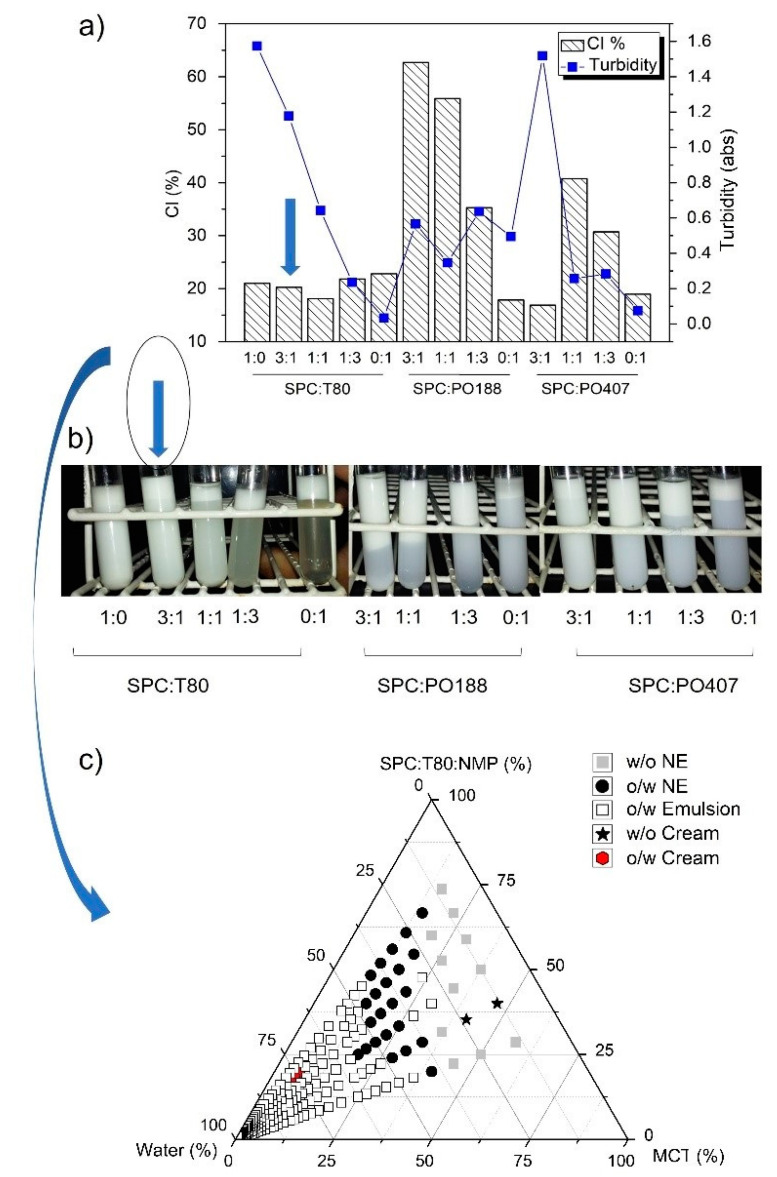
Formulation studies for assessing the surfactant composition: SPC:T80, SPC:PO188, SPC:PO407. (**a**) Creaming index (CI) and Turbidity of different emulsions; (**b**) Images of each tested formulation; (**c**) Pseudo-ternary phase diagram using the SPC:T80 (3:1 *w*/*w*).

Several surfactant mixtures were evaluated to better stabilize the emulsion samples. Different compounds were tested. Polysorbate 80, poloxamer 188, and poloxamer 407 as hydrophilic surfactants, and soy phosphatidylcholine (SPC) as hydrophobic surfactant. Figure 1a,b show the creaming index and the turbidity of the emulsions, respectively, for the systems prepared by using the different surfactants pairs.

The creaming index increase recorded from different samples represent an instability phenomenon. The turbidity of non-creamed phase (lower phase) was also measured to evaluate the ability of the tested surfactant pair to retain the oil dispersed in the system. With an increasing ratio of hydrophilic surfactants, the nanoemulsions became more unstable, with higher creaming index (Figure 1a) and less turbid aqueous phase (Figure 1b). Based in this initial trials, three systems, using SPC only, SPC:T80 (3:1 *w*/*w*) and SPC:PO407 (3:1 *w*/*w*) as surfactants, respectively, were selected as the most stable for conducting further studies. The addition of three co-solvents, such as N-methyl-pyrrolidone (NMP), glycerol, and propylene glycol, was tested to improve the stability of the nanoemulsions and, therefore, decrease the creaming phenomenon. However, NMP was the only one capable to dissolve the SPC surfactant. The initial trials of formulations revealed that the use of SPC only, as a surfactant, even associated with NMP was unable to producing stable nanoemulsions and the phase separation occurred rapidly.

Figure S1 shows the effect of NMP addition on the creaming index and turbidity of selected nanoemulsions, demonstrating that the mixture of SPC:T80 (3:1 *w*/*w*) produced more stable system than SPC:PO407 (3:1), which was selected for further studies using the PPTD (Figure 1c). This assay was conducted under heating (70 ± 2 °C). The NMP was used as co-solvent at the SPC:T80:NMP 3:1:3 (*w*/*w*/*w*) ratio. Some points of o/w nanoemulsions containing considerable amounts of surfactants are observed in the PPDT (Figure 1c), but no transparent or less turbid liquid crystal was observed.

**Figure 2:**
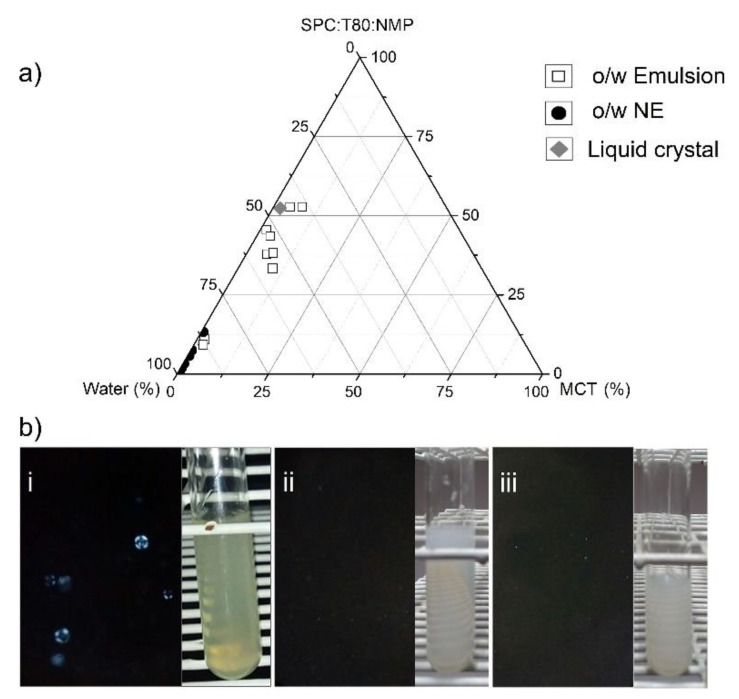
Phase diagram constructed at 25 °C, followed by ultra-turrax homogenization (11,000 rpm). (**a**) Pseudo-ternary phase diagrams (PTPD). (**b**) Polarized microscopy images (**left side**—increased 400×) and visual aspect (**right side**) of (**i**) gel-like system, (**ii**) anionic NE and (**iii**) cationic NE-PEI.

Consequently, the amount of NMP have to be enhanced in the mixture SPC:T80:NMP 6:2:15 (*w*/*w*/*w*), as a tentative to explore a second PPDT constructed without heating at 25 ± 2 °C (Figure 2a). Among the few points explored to produce O/W systems, it was observed not only emulsions and nanoemulsions, but also a less turbid gel like sample similar to a liquid crystal. The gel-like sample containing 2% *w*/*w* of oil phase was identified as an anisotropic phase. The Maltese crosses in Figure 2bi suggest a lamellar-like liquid crystal. The amount of used NMP was able to dissolve SPC at 25 °C without heating. The dilution of this sample with purified water formed liquid and translucent colloidal dispersions containing 0.9% *w*/*w* of oil phase Figure 2bii. The same occurred when the dilution occurred with PEI aqueous solution Figure 2biii. The completely dark polarized microscopy images confirmed the expected isotropic behavior for the nanoemulsions. It is interesting to observe that the amount of both surfactant and the co-solvent were half-reduced in the NE formulations.

The Table 1 shows the composition of systems presented in the Figure 2. The two formulations (NE and NE-PEI) were selected for TsV loading and further evaluation of their physicochemical properties and the in vitro/in vivo performance.

### 3.2. Physicochemical Properties of Anionic and Cationic NE Formulations

**Figure 3:**
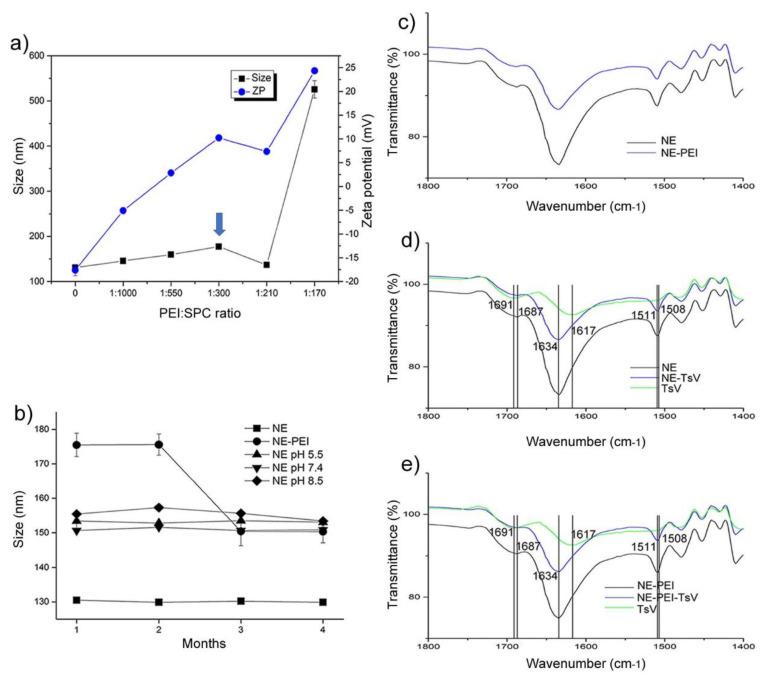
Effect of PEI covering and TsV-loading on the physicochemical properties of the NE formulations. (**a**) Effect of PEI content on the size and zeta potential (ZP); (**b**) Stability and robustness of NE from pH changes; (**c**–**e**) Effect of the composition on the FTIR spectra of distinct NEs: (**c**) PEI addition; (**d**) NE-TsV loading; (**e**) NE-PEI-TsV-loading.

The same approach used to obtain the anionic NEs from the liquid crystal was used to produce the cationic NE-PEI. The selected PEI:SPC ratio have achieved a positive zeta potential in the tested formulations (Figure 3a). The experimental data revealed that the PEI:SPC = 1:300 *w*/*w* induced cationic-covered NE-PEI with zeta potential of approximately +10 mW with droplet size < 200 nm. Formulations with superior PEI:SPC ratio were not selected because the well-established toxicity of PEI or enhancement of the droplets size. The Figure 3a shows the data from physical stability assay, adequately presented in the Section 3.3.

FT-IR spectra recorded for different NE formulations are shown in Figure 3c–e. The cationic covering NE-PEI reduced the intensity of the FTIR bands related to the anionic NE (Figure 3c). This achievement suggests that PEI anchoring perturbs the surface of NEs. The same effect occurs for the TsV-loaded formulations (Figure 3d,e). In addition, it was possible to observe the bathochromic shift of the band recorded in the region of 1634 cm^−1^ in the NE for 1617 cm^−1^ after TsV-loading for both NE and NE-PEI. This fact suggests relevant intermolecular interactions among the proteins from TsV with PEI on the surface of NEs. Generally, the amide I C-N and amide II C-N stretch are recorded for proteins at approximately 1600–1700 cm^−1^ and 1500–1600 cm^−1^, respectively.

### 3.3. Physical Stability of NE Formulations Against pH and Saline Content

The NE formulations discussed in this study were designed as biocompatible adjuvants for ready venom-loading by adsorption on surface of droplets, as performed in antiserum production. Thus, the stability of TsV was not considered, but only the ability of nanocarrier resists to possible stress steps involved, such as possible ionic strength or pH changes. Since that SPC is an amphoteric surfactant and that the PEI affects the size of the NEs, the robustness of the NEs to resist pH adjustment and effect of PEI covering on the physical stability were both assessed for four months (Figure 3b). The pH adjustment of the NE formulations was performed using PBS with titrations of different amounts of HCl 0.1 M^−1^ or NaOH 0.1 M^−1^ aqueous solutions. The results revealed that the pH variation did not affected the NE stability. Table S1 shows detailed values of size, PdI, and zeta potential measured for studied formulations for entire interval (four months).The presence of PEI promoted an increase in droplet size from 125 to 175 nm, which dropped to 150 nm after the 3rd month. The decrease of droplet size of about 175 nm (after two months) to 150 nm (after three months) can be statistically different, but we have not considered a relevant change able to affect the physical stability of colloidal dispersions and its performance. This achievement is considered a ripening process, mainly considering the PEI equilibrium between the surface/interface of droplets and aqueous phase.

After assessing the physical stability of different NE formulations, the TsV-loading was performed for NE and NE-PEI samples. These four formulations remained stored at 4 °C for six weeks and their physicochemical properties were also evaluated at the final interval, as showed in Table 2. The experimental data revealed that both the cationic character and the physical stability were preserved. Indeed, no considerable effect on the droplet size or zeta potential was observed. The PdI values of all nanoemulsions stored at 4 °C (Table 2) are lower than the PdI stored at 25 °C (Supplementary Table S1). This fact may be explained due to the expected changing in the solubility of surfactants at distinct temperatures, which affects the self-assembly behavior. However, the formulations preserved the uniform droplet size when stored at 4 °C. The Figure S2 illustrates some examples. In fact, for the NE-PEI stored for four months, the PdI also ranged from 0.22 to 0.18, values inferior to 0.3. The zeta potential of NE-PEI also slightly decreases over time due to any rearrangement of anchored PEI on the surface of NE, altering the PEI ratio dispersed in the aqueous phase. This achievement corroborates with the observed decrease in droplet size, after four months.

### 3.4. In Vitro Cell Viability and Hemolytic Tests

**Figure 4:**
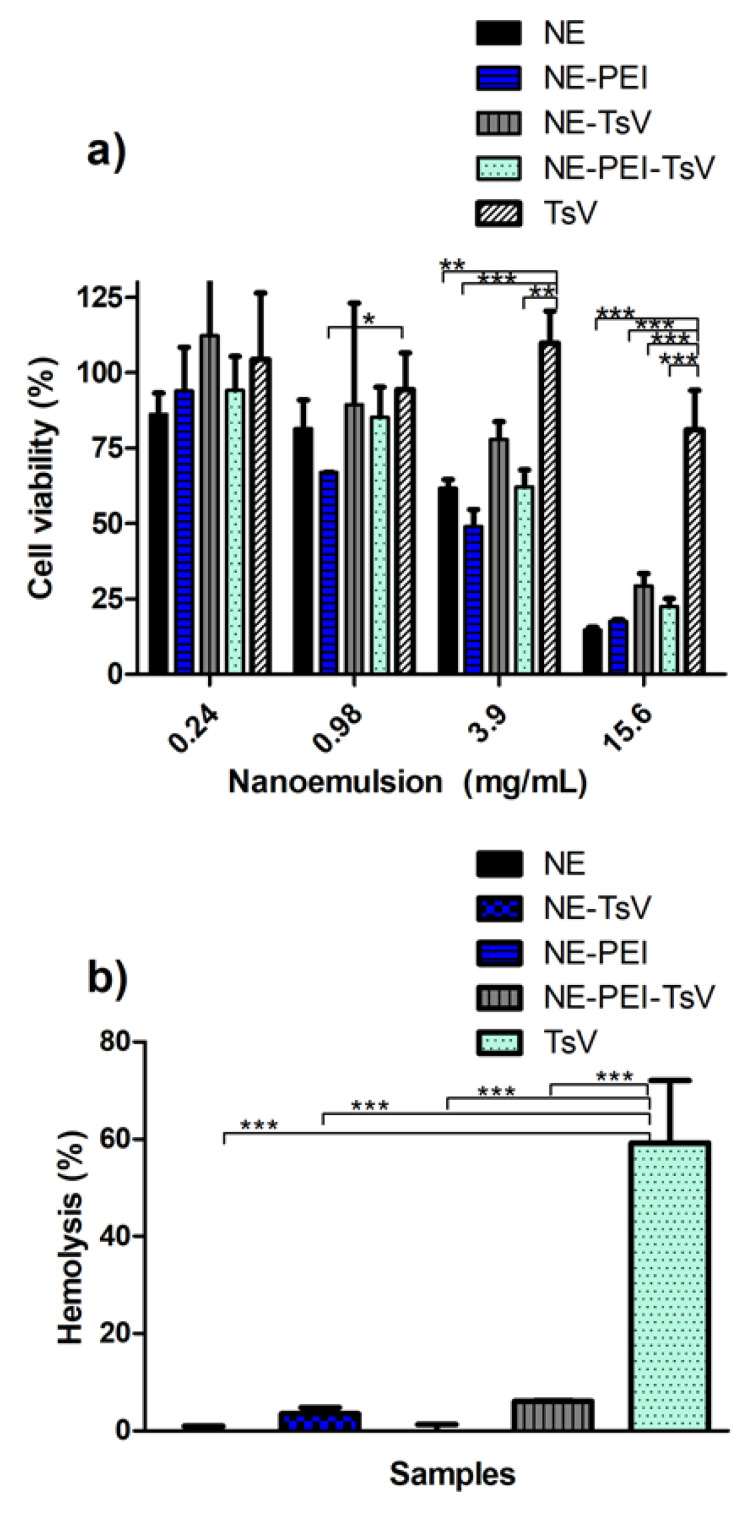
(**a**) Cell viability test on RAW 264.7 cells against different formulation and TsV alone. (**b**) Hemolysis test on human red blood cells against different formulation and TsV alone. The comparisons were performed using the one-way ANOVA followed by the Tukey’s post-test. A *p* < 0.05 was represented as *, *p* < 0.01 as ** and *p* < 0.001 as ***. Note: TsV = 0.018 mg/mL for loaded nanoemulsions and TsV alone.

The TsV-loaded NE formulations have a final TsV concentration of 0.018 mg/mL, which make almost impossible to evaluate, with precision, its in vitro release behavior due to the small amount of venom. Thus, in vitro cell viability and hemolytic tests were performed for evaluating, indirectly, the different behavior profiles of the TsV release and their interactions with the cell in the biological medium. Moreover, comparisons among different TsV containing formulations and TsV alone were performed. The NE, NE-PEI and respective TsV-loaded formulations were subjected to the cell viability tests in the murine RAW 264.7 cell line (Figure 4a). The results revealed a dose dependent cytotoxicity. However, all NE formulations were biocompatible (no cytotoxic) in a concentration equal or less than 0.98 mg/mL, except for the NE-PEI. At high concentration (above 0.98 mg/mL), all formulations induced comparable cytotoxicity.

Since that hemolytic effect of TsV is well established in the literature, the ability of NEs to reduce or impairs this effect was also tested (Figure 4b). The hemolysis test confirmed the biocompatibility of both NE and NE-PEI formulations. In addition, comparisons of TsV-loaded NEs formulations with the same concentration of TsV alone (0.018 mg/mL) revealed an extremely low degree of hemolysis from the loaded formulations. This is an important finding, mainly considering the future in vivo use of these formulations.

### 3.5. Antivenom IgG Titration

**Figure 5:**
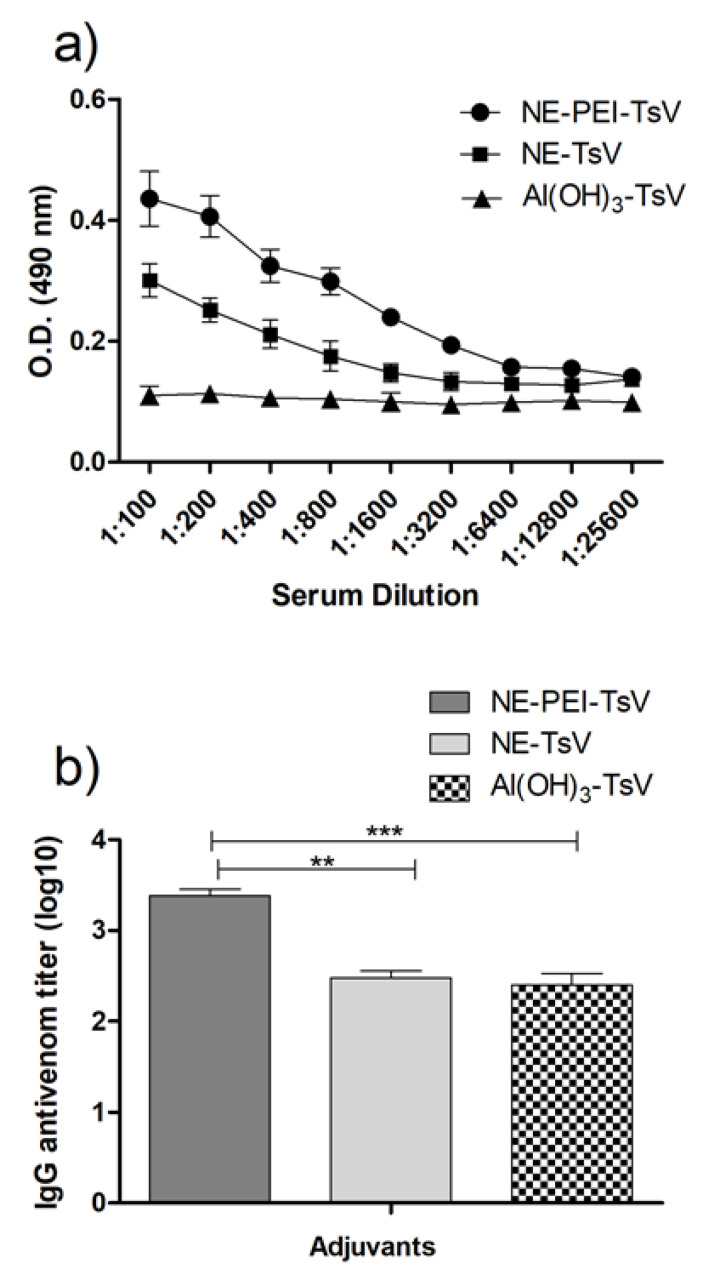
(**a**) Evaluation of the optical density profile from antibodies produced by mice immunized subcutaneously for 6 weeks with 0.0018 mg of TsV using the three tested adjuvants, (**b**) Antivenom IgG titer from mice immunized subcutaneously for 6 weeks with 0.0018 mg of TsV using the three tested adjuvants. The comparisons were performed using the one-way ANOVA followed by Tukey’s post-test. A *p* < 0.05 was represented as *, *p* < 0.01 as ** and *p* < 0.001 as ***.

Figure 5 shows the antibody titer profile produced by the immunized animals with TsV using different adjuvants. The NE-TsV and NE-PEI-TsV were compared to the traditional aluminum hydroxide (Al(OH)_3_) adjuvant. Figure 5a shows the optical density, giving a general view of the specific IgG UV-Vis absorption. It is possible to observe that both NE and NE-PEI exhibited superior density of immunoglobulins compared to the Al(OH)_3_ adjuvant, with greater response for the NE-PEI. The Figure 5b shows the titer measurements and statistics comparisons. The animals immunized with NE-PEI produced statistically higher IgG titer compared to that immunized with both NE and the Al(OH)_3_.

## 4. Discussion

Thus, the co-solvent addition in the NE formulations have been described as an interesting and promising way to produce small-droplet sized NEs [48]. Indeed, the co-solvent reduces the oil/water interfacial tension and decrease the segregation grade among the specific non-polar compounds, improving the diffusion rate of surfactants to the aqueous phase, at the phase inversion moment [35,48,49]. Therefore, the *N*-methyl-pyrrolidone (NMP) was used as a co-solvent in this study due to its ability to dissolve SPC and improve its miscibility in the oily phase of the NEs. NMP has a octanol/water partition coefficient of 1.21, which allows solubilizing several hydrophobic components using this solvent [50]. In this study, the addition of NMP allowed to stabilize the NEs produced with SPC:T80 at 3:1 *w*/*w* ratio. Consequently, this composition was selected to build a second pseudo-ternary phase diagram (Figure 2a). The mixtures prepared with SPC:PO407 at 3:1 w/w ratio showed instability after the NMP addition. The pure SPC was also tested with NMP, but the instability represented by the phase separation remained as the main barrier.

Stable self-assembled NEs can be produced by using a fine balance among the aqueous phase, oil phase, and surfactants. In this study, the SPC was mixed with different surfactants in an initial trial to produce a biocompatible blend able to stabilize MCT (oil phase) in water (Figure 1a) and produce a NE system. The systems prepared with polysorbate (T80) and two poloxamers (PO188, PO407) showed the lowest creaming index for mixtures composed of SPC:T80 at 3:1 *w*/*w* ratio and SPC:PO407 at 3:1 *w*/*w* ratio (Figure 1b). These compositions are interesting and promising for producing pharmaceutical emulsion systems. Indeed, the greater SPC amount (75% *w*/*w*) in the surfactant mixture certainly produces more biocompatible emulsion systems [35,47]. However, produce self-assembled NEs using SPC is not an easy task. Some properties of the SPC, such as melting point, water solubility, and miscibility with oil phase (MCT) at room temperature make this a practically impossible task when low-energy emulsification methods are used.

All the achieved NE formulations pointed out in the PPTD exhibited surfactant content superior to 30% *w*/*w* (Figure 1c). This amount of surfactant is an important obstacle to produce biocompatible and non-hemolytic NEs [35,48,49]. In addition, these formulations showed PdI values >0.4 (data non showed). In addition, no liquid crystal system was observed on the PPTD for the tested compositions. Some previous trials at our laboratory revealed that the temperature perturbed the droplet size and surfactant arrangement of lipid based dispersed systems (data non showed). Hence, the same experiment with the phase behavior diagram was performed at 25 °C. The second diagram revealed an interesting point, a translucent formulation with high viscosity due to its high surfactant content. (Figure 2a). This feature is uncommon to the NEs, but highly likely to be observed in lyotropic liquid crystal systems [35,48,49]. This phenomenon was better characterized using polarized microscopy images (Figure 2bi), which showed Maltese crosses, a characteristic of lamellar liquid crystal structures [51].

In previous studies, our team has already assessed the phase transition of lyotropic lamellar liquid crystals as a strategy to obtain small and uniform droplet sized NEs [26,35]. The self-assembly behavior of surfactants in the oil/water interface depends on a fine and limited phase equilibrium. An optimum water addition could induce phase transitions to NEs. The suitable dilution of the gel-like system identified in the second diagram corroborated to this assumption in Figure 2bii. Furthermore, this method produced small droplet sized NE formulations with remarkable low PdI values (Table 2).

The continuous addition of water in the lamellar mesophases can produce intermediate bicontinuous microemulsions with final transition to the NEs [26,33]. A similar approach considered a phase transition from cubic liquid crystals to NEs [51,52]. The dilution of cubic liquid crystal also produced small droplet-sized NEs compared to other techniques, such as high strength or ultrasonication [51]. Unfortunately, these authors diluted the cubic mesophases using the hexadecane as a co-solvent, which is not suitable for application in pharmaceuticals. Thus, the liquid crystal dilution technique presented in this study allows the production of NEs with desirable properties using pharmaceutical grade ingredients. Moreover, the final NEs produced by this approach were subjected to a careful physicochemical characterization for further TsV loading and use of such formulations in in vivo and in vitro studies.

The pharmaceutical application of NEs as drug delivery systems relies on their ability for solubilizing drugs in the oil or o/w interface region. The rationale behind this study was to hypothesize that proteins such as the ones found in the TsV could be adsorbed on the NE surface by hydrophobic or electrostatic anchoring. Previous studies have reported the anionic character of TsV at physiological pH and the successful TsV entrapment into the cationic chitosan-based nanoparticles [53]. Similar approaches with venom from different scorpion and snake species reported the venom-loading by adsorption on the cationic surface of nanoparticles [54,55,56]. However, the zeta potential of the NE prepared by diluting the liquid crystal identified in the PPTD (Figure 2a) was anionic (Table 2). This limitation was solved by adsorption of the cationic macromolecule PEI. A specific PEI aqueous solution was used in the dilutions, generating NE-PEI systems (Figure 2biii). The minimum PEI:SPC ratio able to induce stable cationic NE with a minimal increment on droplet size was carefully assessed (Figure 3a). The PEI:SPC ratio of 1:550 *w*/*w* produced NE with zeta potential close to zero, and droplet size of about 150 nm. The chosen PEI:SPC ratio of 1:300 *w*/*w* was able to produce translucent NEs (Figure 2biii). Similar approaches have demonstrated the use of polymers and macromolecules for stabilizing emulsion systems [57,58], but the high surfactant concentrations remains as the main barrier for their application as parenteral pharmaceutical emulsions. In this study, we found interesting NEs containing of approximately 8% w/w of surfactant mixture (Table 1) in which 75% is SPC, a biocompatible surfactant used in parenteral nutrition formulations.

Since that SPC is an amphoteric surfactant and that the PEI arrangement on the surface of NEs can be affected by the pH of medium, the formulations were subjected to a stability test performance using different aqueous media (Figure 3b). The cationic NE-PEI and anionic NEs were stable for the entire period interval of four months for all tested conditions, which included physiological PBS, acid, alkaline, and neutral pH. This assay has shown the robustness of the NE-PEI to be used for protein-loading, which in most cases requires some pH adjustment or dilution of the final formulation. Generally, the addition of saline solutions in the NEs induces droplet size increase because the expected increasing in the Debye length [59]. In this study, it was observed a slight increase of about 25nm for the NE systems (Figure 3b). In addition, the NE-PEI sample presented a slight decrease in size and zeta potential after three months (Figure 3b and Supplementary Table S1). However, the PdI also ranged from 0.22 to 0.18. This fact suggests that anchored PEI on the surface of NE droplets is in a constant equilibrium with PEI dispersed in the aqueous phase, as a ripening process. Considering the TsV-loaded NEs, the anchored protein on the NE droplets also may be at balance with PEI-protein aggregates. It is interesting to reinforce that the venom storage as a biologic material is a limitation for long-term storage. Thus, the easily and simple preparing of NE and cationic-covered NE by diluting the LC followed by venom-loading for the intend application is a promising strategy considering the commercially available immunizing protocols. The TsV-loading maintained important physicochemical properties of stable and reproducible colloidal systems as small size and low PdI values (Table 2).

*Tityus serrulatus* venom is a complex mixture of proteins, peptides, polysaccharides, and others. Their supramolecular binding on the surface of the PEI covered NE could allow a slow release of venom enzymes and antigens for a controlled and protective response of the immune system, as similarly reported for cationic nanoparticles [60]. In this context, the interactions among the compounds of the formulations were further investigated by FT-IR spectroscopy (Figure 3c–e). This experiment followed the additional covering of the anionic NE formulation identified in the second PPTD (Figure 2b). Thus, the Figure 3c shows the effect of the PEI covering on the NEs, reducing the intensity of their FT-IR bands. This fact can be better observed for the characteristic C=O stretch at 1634 cm^−1^ of the carbonyl presented in both the used surfactants and the MCT oil phase. The same effect was observed for the NE-TsV (Figure 3d). In addition, the primary amide bands could be observed in the range of 1600–1700 cm^−1^, such as that at 1600–1639 cm^−1^ for the β-sheet, as well as at 1651–1660 for the α-helix portion, and at 1661–1700 cm^−1^ for the T-turns [61,62]. Considering the TsV-loaded cationic covered NEs in the Figure 3e, the absorption band of the amide I of proteins from TsV can be seen at 1617 cm^−1^. The equivalent band was also highlighted at 1634 cm^−1^, the same observed for the anionic NE systems. This phenomenon suggests that a possible hydrophobic interaction with the β-sheet portion of the proteins from the TsV preserved the initial conformation. The hypsochromic shift from 1617 cm^−1^ to 1634 cm^−1^ is considered within β-sheet conformation range. In addition, the Figure 3c,d show a bathochromic shift of absorption bands from 1692 cm^−1^ to 1687 cm^−1^. These results corroborate to the expected T-turns conformation of some proteins. Summarizing, some conformational changes in the β-sheet and T-turns regions occurred after TsV-loading, but those changes remained within the same secondary fold region in the protein structure. Similar achievements were reported in the literature when the lysozyme was adsorbed on the surface of Fe_2_O_3_ nanoclusters [61]. Thus, the experimental FTIR spectroscopy results provided valuable information to understand the interactions of TsV on the surface of NEs, mainly regarding the followed steps of PEI covering of NEs and additional TsV-loading.

The cell viability experiments showed a dose dependent cytotoxic effect of the NE formulations (Figure 4a). This result highlights the statistical differences between venom solution (with extremely low concentration, 0.018mg/mL) with the formulations, which ranges of 0.24 to 15.6 mg/mL, which was 13 to 850 over superior to that used for TsV solution. It was not observed statistical differences between TsV-loaded NE and cationic-covered NE formulations. This fact suggests that the selected PEI:SPC at 1:300 *w*/*w* ratio for the NE-PEI did not increased the toxicity of the formulation compared to NE formulation itself. Moreover, at 3.9 mg/mL, the cell survival rate was about 60% and 50% for the NE and the NE-PEI, respectively. The respective TsV-loaded samples showed a trend of superior cell survival rates. This results also reinforces the importance of exploring the concentration range of PEI able to supply an expected cationic charge, with a minimal cytotoxicity effect compared with non-cationic NE formulation. These results are promising for further in vivo evaluation of the selected formulations as adjuvants.

We considered that NE formulations discussed in this study are suitable for in vivo application. Several reported studies with nanoemulsions subjected to viability studies revealed to be more cytotoxic or similar than that demonstrated in the present study [63,64,65]. We recognize that it is not a comparison between formulations with only one variable. The cell lines and nanoemulsion composition should be considered as important variables. Since that nanoemulsions present oil droplets covered with surfactants in aqueous media, the cytotoxicity is generally connected with type and concentration of surfactants [66,67].

Regarding the TsV activity, some physiological responses have been demonstrated in rats, such as dehydration, hemoconcentration, complement system activation, hepatic and lungs inflammation, and hemolysis [68,69]. Thus, a hemolytic assay was conducted for all tested formulations (Figure 4b). Contrary to the cell culture experiments, the TsV have shown a considerably hemolytic effect at the same 0.018 mg/mL concentration previously used for the cell viability experiment. The Ca^2+^ influx was previously reported as the main mechanism responsible for the venom hemolytic activity, which activates phospholipases that act in the cell membrane [68]. However, recent studies demonstrates that TsV does not activate phospholipases [70], suggesting an alternative mechanism that causes hemolysis. Nevertheless, both NE and NE-PEI were able to reduce this hemolytic effect referred to TsV. The experimental data suggests that a possible supramolecular aggregation of TsV on the NE surface avoids this harmful effect, an important feature for further in vivo experiments.

Finally, the in vivo potential of the loaded NE and NE-PEI as adjuvants for antiserum production against TsV was evaluated and compared to the Al(OH)_3_ adjuvant. Figure 5a showed the evaluation of antibody profile for the animals immunized with each sample. Differences can be observed among the optical density observed for the three formulations. As hypothesized, this experiment corroborated to the expected improved adjuvant activity for the TsV-loaded NEs. A superior performance was observed for the NE and NE-PEI compared to the Al(OH)_3_ traditional adjuvant. (Figure 5a). The Al(OH)_3_ adjuvant is quite potent and used in considerable number of approved vaccines, which still has an adjuvant to be overcome. In addition, aluminum is the most common formulation for serum production in antivenom protocols. We can observe several studies in the literature in which the response of nanocarrier was not better that aluminum salt or should be associated with it to induce a similar response. In a previous study, we have demonstrated that chitosan NPs was capable to induce a statistically similar IgG titer to Al(OH)_3_, but not superior [53]. Thus, not all nanocarriers are capable of enhance IgG titers, which for antivenon serum are essential for neutralizing the toxins. Nanocarriers as adjuvants may act, in some cases, stimulating the immune response by different mechanism other that immunoglobulins production. Therefore, the adjuvant response depends not only on the use of nanocarriers, but by on their size, shape, hydrophobicity, and charge of particles, which can be tailored.

Additionally, Figure 5b shows that the NE-TsV induced a similar IgG titers response to the animals exposed to Al(OH)_3_. This result demonstrates the superior performance expected for the cationic NE-PEI compared to the anionic NE system. Since that aluminum salts are approved and are one of the most used adjuvants for antiserum production [71,72], the NE-PEI formulation address a promising and novel device to this purpose. In addition, the aluminum generally induces a major production of nonspecific antibodies against the toxins, causing several side effects [73]. The larger IgG titers produced by the animals treated with NE-PEI-TsV suggests that this is a promising adjuvant against the harmful scorpion toxins, and, probably, is able to induce a more effective antivenom serum. The superior IgG titers of developed nanocarriers confirms its delivery effectiveness for venom immunogens, possibly increasing cell recognition or even cell uptake of these immunogens. Vaccine field may be benefitted of this type of nanocarriers adjuvants, as literature investigations demonstrated for immunogens and also nanocarrier association with immune agonists adjuvants to potentialize the effect associating two adjuvants mechanisms [14,15,16]. The PEI-covered nanoemulsions with superior IgG stimulant production showed that this biocompatible polymer capable of enhance cell uptake of nucleic acids and drugs [42,43,44] also may enhance protein cell recognition and be applied as vaccine adjuvant.

A critical discussion about the anti-serum therapy is necessary to achieve new adjuvants able to induce an efficient immune response with minimal side effects [70]. The aluminum salts were introduced as adjuvant in 1920s decade, and this class of compounds are used in most of 140 approved vaccines for the prevention of diseases [70]. However, the inflammation problems associated to the aluminum salts as adjuvants are well reported in the literature, which include neurotoxicity. Furthermore, the majority of antibodies in antiserum produced by the immunization of host animals with traditional adjuvants are not capable to neutralize the venom toxins [70]. Thus, the NEs platform discussed in this study provide biocompatible formulations able to be used as adjuvant for antiserum productions against TsV, with superior performance compared to the tested aluminum compound.

Additionally, the experimental data corroborates that supramolecular aggregation of TsV on the surface of NE did not decrease the recognition of the epitopes. In fact, the increasing polyclonal IgG antibodies titers could improve antivenom serum efficacy and improve the performance of TsV, which could enable a possible lower TsV dose in the final adjuvant formulation.

## 5. Conclusion

For the first time, different formulations of self-assembled biocompatible NEs were designed as a delivery system for antigens of the *Tityus serrulatus* scorpion venom. Several NE compositions were carefully assessed using a PPTD approach. The dilution of the gel-like liquid crystal induced the formation of small droplet-sized and stable anionic nanoemulsions. The dilution with a specific and optimized PEI solution produced cationic covered NEs. The in vitro experiments showed that both anionic and cationic NEs were able of TsV-loading, which have shown low cytotoxicity and low hemolytic effect. Finally, the ability of NE systems as adjuvant was tested in Balb-C mice and compared to the traditional Al(OH)_3_ adjuvant. Possibly the NE systems increases the cell recognition or even cell uptake of these immunogens. The adjuvant performance of the cationic NE formulation was superior to that of the Al(OH)_3_ adjuvant. In addition, the antivenom IgG titer was considerably enhanced in animals immunized with TsV-loaded cationic-covered NE. In this study, we demonstrated that is possible to modulate the physical chemical properties of self-assembled NEs to induce different performances as adjuvants. As hypothesized, these colloidal nanocarriers are promising adjuvants for antiserum production against the *Tityus serrulatus* scorpion venom.

## Figures and Tables

**Table 1:** Composition of the formulations.

Ingredient Amount	Formulations				
	Gel-Like System	Blank Anionic NE	Tsv-Loaded Anionic NE	Blank Cationic NE	Tsv-Loaded Cationic NE
TsV (µg/mL)	0.0	0.0	18.0	0.0	18.0
MCT (% *w*/*w*)	2.00	0.9	0.9	0.9	0.9
T80 (% *w*/*w*)	4.55	2.0	2.0	2.0	2.0
SPC (% *w*/*w*)	13.65	6.0	6.0	6.0	6.0
NMP (% *w*/*w*)	34.2	15	15	15	15
PEI (% *w*/*w*)	0.0	0.0	0.0	0.02	0.02
Water (% *w*/*w*)	45.6	76.1	76.1	76.08	76.08

Notes: TsV=*Tytyus serrulattus* venom; MCT = medium chain triglycerides oil; T80 = polysorbate 80; SPC = soy phosphatidylcholine; NMP = N-methyl-pyrrolidone; PEI = poly(ethyleneimine).

**Table 2:** Droplet size and zeta potential measurements for different blank and TsV-loaded NEs stored in hermetically sealed vials at 4 °C, for six weeks.

Formulation	Mean Size	Pdi	Zeta Potential (Mv)
NE	125.7 ± 0.3	0.13 ± 0.03	−18.3 ± 1.0
TsV-loaded NE	126.3 ± 1.3	0.13 ± 0.01	−13.3 ± 0.6
NE-PEI	165.2 ± 0.5	0.13 ± 0.01	8.4 ± 1.4
TsV-loaded NE-PEI	167.1 ± 0.5	0.13 ± 0.01	5.04 ± 0.09

## Supplementary Materials

The following are available online at https://www.mdpi.com/1999-4923/12/10/927/s1, Figure S1: Effect of N-methyl-pyrrolidone (NMP) in association with mixtures of soy phosphatidylcholine (SPC) with The polysorbate 80 (T80) or poloxamer 407 (PO407) on the creaming index of emulsions of medium-chain triglyceride (MCT) in water, Figure S2: Droplet size distribution (zeta-sizer images) with respective PdI and zeta potential values assessed for the studied formulations, Table S1: Size, PdI, and Zeta potential measurements for different formulations, at distinct intervals of physical stability of NE formulations against pH and saline content.
