# Supplementary material for: Self-Assembled Cationic-Covered Nanoemulsion as A Novel Biocompatible Immunoadjuvant for Antiserum Production Against Tityus serrulatus Scorpion Venom
Source: Pharmaceutics. 2020 Sep 29;12(10):927. doi: 10.3390/pharmaceutics12100927 (PMC7599857; doi:10.3390/pharmaceutics12100927)
Supplement: Supplementary file 1 [file pharmaceutics-12-00927-s001.pdf]

Self-Assembled Cationic-Covered Nanoemulsion as A Novel Biocompatible Immunoadjuvant for Antiserum Production Against *Tityus Serrulatus* Scorpion Venom

Arthur Sérgio Avelino de Medeiros, Manoela Torres-Rêgo, Ariane Ferreira Lacerda, Hugo Alexandre Oliveira Rocha, Eryvaldo Sócrates Tabosa do Egito, Alianda Maira Cornélio, Denise V. Tambourgi, Matheus de Freitas Fernandes-Pedrosa and Arnóbio Antônio da Silva-Júnior

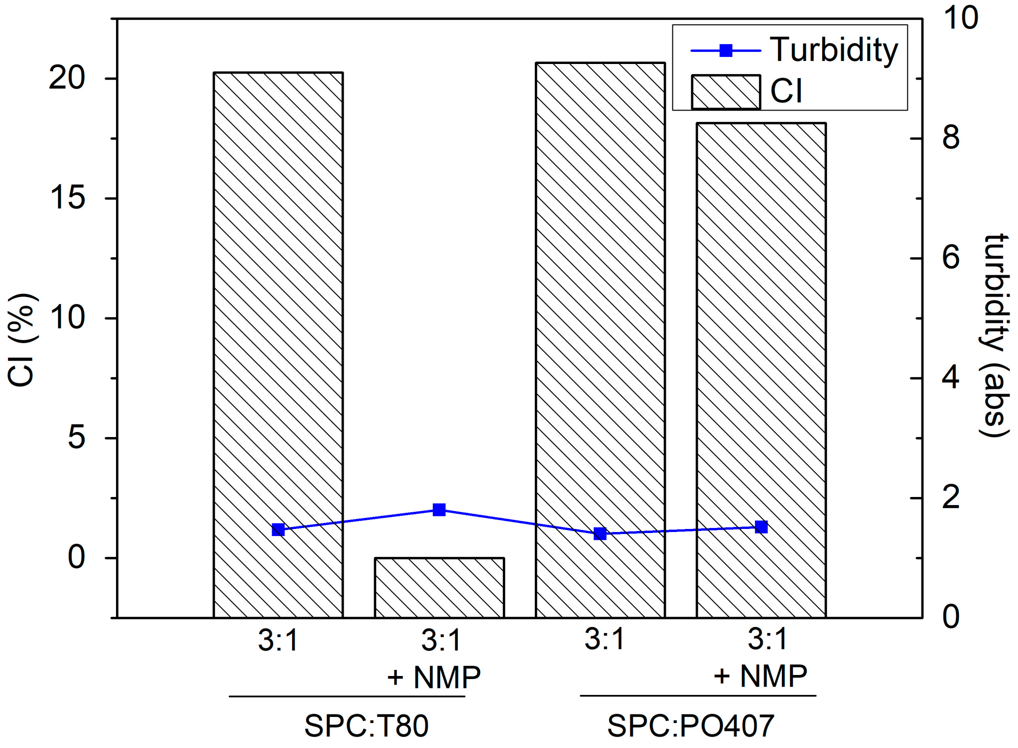

Figure S1. Effect of N-methyl-pyrrolidone (NMP) in association with mixtures of soy phosphatidylcholine (SPC) with The polysorbate 80 (T80) or poloxamer 407 (PO407) on the creaming index of emulsions of medium-chain triglyceride (MCT) in water.

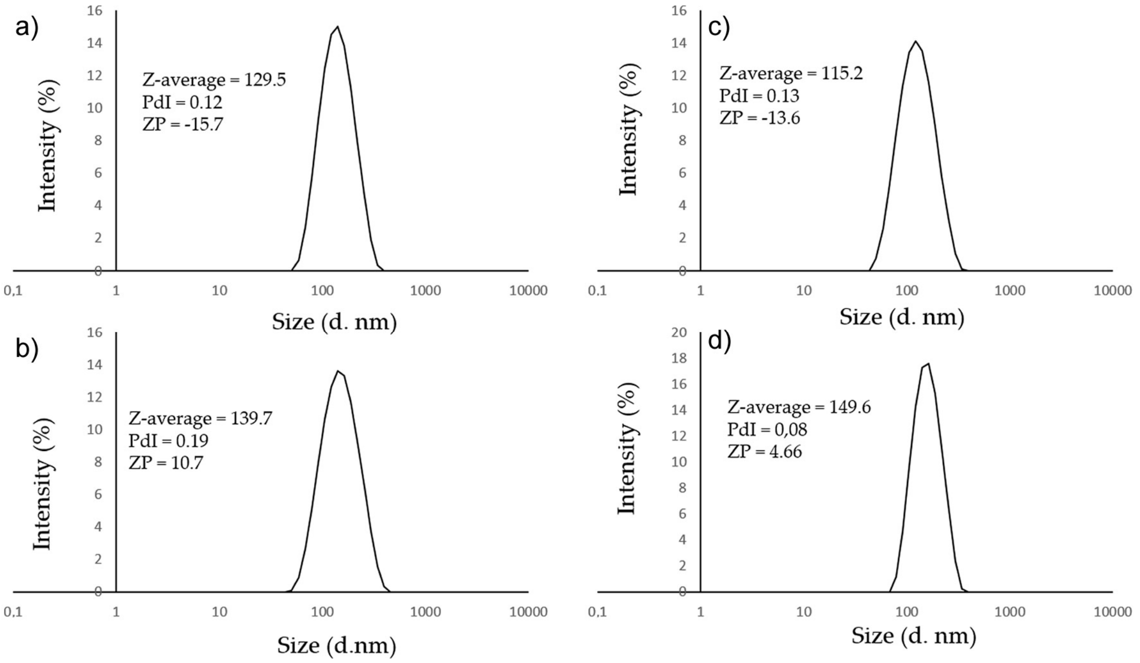

Figure S2. Droplet size distribution (zeta-sizer images) with respective PdI and zeta potential values assessed for the studied formulations: (**a**) nanoemulsion (NE) and (**b**) cationic-covered nanoemulsion NE-PEI after 24 hours of preparing stored at 25 °C (**c**) TsV-loaded NE and d) TsV-loaded NE-PEI after 6 weeks, stored at 4 °C storage.

Table S1. Size, PdI, and Zeta potential measurements for different formulations, at distinct intervals of physical stability of NE formulations against pH and saline content .

**Samples**	**Measurements of size (nm), PdI, and zeta potential (mV) at distinct intervals**			
	1month	***2 months***	***3 months***	***4 months***
	130.73 ± 0.38	130.50 ± 0.38	129.90 ± 0.40	130.20 ± 0.74
NE	0.14 ± 0.02	0.14 ± 0.02	0.14 ± 0.02	0.13 ± 0.02
	−17.6 ± 1.20	−17.6 ± 1.30	−16.7 ± 1.50	−16.4 ± 1.20
	153.50 ± 0.8	153.40 ± 0.09	152.80 ± 0.05	153.06 ± 0.65
NE pH 5.5	0.07 ± 0.01	0.09 ± 0.01	0.1 ± 0.01	0.1 ± 0.01
	−16.3 ± 1.30	−15.6 ± 0.35	−16.5 ± 0.29	−10.6 ± 0.25
	150.65 ± 0.43	151.6 ± 0.55	150.6 ± 0.45	150.8 ± 0.85
NE pH 7.4	0.1 ± 0.003	0.09 ± 0.005	0.08 ± 0.008	0.11 ± 0.02
	−15.3 ± 0.25	−14.7 ± 0.32	−15.6 ± 0.34	−12.9 ± 0.36
	155.4 ± 0.85	157.3 ± 0.5	155.6 ± 0.4	153.4 ± 0.8
NE pH 8.5	0.13 ± 0.003	0.14 ± 0.002	0.13 ± 0.004	0.10 ± 0.009
	−14.2 ± 0.65	−13.6 ± 0.35	−13.5 ± 0.35	−9.5 ± 0.35
	175,5 ± 3.4	175,6 ± 3.1	150,4 ± 4.1	150.3 ± 3.2
NE-PEI	0.24 ± 0.02	0.21 ± 0.02	0.22 ± 0.02	0.18 ± 0.02
	10.76 ± 0.65	7.76 ± 0.54	4.45 ± 0.34	0.56 ± 0.09
